# High-dimensional Metaverse Platforms and the Virtually Extended Self

**DOI:** 10.5334/joc.327

**Published:** 2024-01-09

**Authors:** Thomas D. Parsons

**Affiliations:** 1 Grace Center, Edson College, Arizona State University, Tempe, AZ, US; 2 Computational Neuropsychology & Simulation (CNS) Lab, Arizona State University, Tempe, AZ, US

**Keywords:** Metaverse, Cognition, Extended Reality, Extended Cognition, Ecological Validity

## Abstract

The study of cognition has traditionally used low-dimensional measures and stimulus presentations that emphasize laboratory control over high-dimensional (i.e., ecologically valid) tools that reflect the activities and interactions in everyday living. Although controlled experimental presentations in laboratories have enhanced our understanding of cognition for both healthy and clinical cohorts, high dimensionality may extend reality and cognition. High-dimensional Metaverse approaches use extended reality (XR) platforms with dynamic stimulus presentations that couple humans and simulation technologies to extend cognition. The plan for this paper is as follows: The “Extending from low to high-dimensional studies of cognition” section discusses current needs for high-dimensional stimulus presentations that reflect everyday cognitive activities. In the “Algorithmic devices and digital extension of cognition” section, technologies of the extended mind are introduced with the Metaverse as a candidate cognitive process for extension. Next, in the “A neurocognitive framework for understanding technologies of the extended mind” section, a framework and model are proposed for understanding the neural correlates of human technology couplings in terms of automatic algorithmic processes (limbic-ventral striatal loop); reflective cognition (prefrontal-dorsal striatal loop); and algorithmic processing (insular cortex). The algorithmic processes of human-technology interactions can, over time, become an automated and algorithmic coupling of brain and technology. The manuscript ends with a brief summary and discussion of the ways in which the Metaverse can be used for studying how persons respond to high-dimensional stimuli in simulations that approximate real-world activities and interactions.

## 1. Extending from Low to High-Dimensional Studies of Cognition

### 1.1 Ecological Validity versus Experimental Control

Ulric Neisser’s (1967) seminal textbook *Cognitive Psychology* urged researchers interested in cognition to study the various processes by which sensory input is transformed, reduced, elaborated, stored, recovered, and used (see also Neisser, 1976, 2007). For Neisser, this approach to cognition included analyses that extended from the perceptual inputs to pattern recognition, as well as memory and recall (Neisser, 2007). Since Neisser’s early suggestions for studying cognition, many clever experimental manipulations have been employed to discover cognitive processes involved when human brains interpret, behave, and interact with their environments. With the advent and development of cognitive neuroscience, there has been an increasing (even if implicit) emphasis on rigorously controlled experimental manipulations that filter out as much noise (e.g., confounding variables) as possible so that true signals can be isolated. As a result, the study of cognition has a long history of tightly controlled laboratory studies using low-dimensional stimulus presentations and tools (i.e., static stimuli; limited interactivity; text-based vignettes). It is important to note that the use of controlled experiments with restricted stimulus presentations (low-dimensional) has allowed for notable advances in our understanding of cognition.

Over the years, researchers of cognition have at times questioned whether limiting the study of cognition to rigorously controlled laboratory experiments offered insight into the high-dimensional phenomena (everyday memory, judgement, and decision making) found in everyday experiences. In response, Neisser delivered a now famous (infamous for some) opening address in 1976 (later published in 1978) to the first International Conference on Practical Aspects of Memory, in which he argued for greater ecologically validity in cognitive assessment. Neisser offered three main challenges to cognition (e.g., memory) research: (1) the lab-based approach has offered few new discoveries; (2) the emphasis on broad theoretical issues neglects questions relevant to everyday life; and (3) the strictly controlled experimental settings are artificial and employ measures that have few counterparts in everyday life. Neisser and several cognitive psychologists who followed (Aanstoos, 1991; Barnier, 2012; Kingstone et al., 2008; Shamay-Tsoory and Mendelsohn, 2019; Osborne-Crowley, 2020) were expressing the view that this level of experimental control and limited stimulus presentation (e.g., word lists and static stimuli). Concerns about studying cognition in controlled experimental presentations of stimuli can be found in many areas: educational psychology (Dunlosky et al., 2009), child development (Lewkowicz, 2001; Schmuckler, 2001; Adolph, 2019), clinical neuropsychology (Chaytor & Schmitter-Edgecombe, 2003; Parsons, 2015; Pinto et al., 2023); cognitive neuroscience (Hasson and Honey, 2012; Maguire, 2012; Hamilton and Huth, 2018; Matusz et al., 2019; Sonkusare et al., 2019), and social neuroscience (Schilbach et al., 2013; Schilbach, 2015; Shamay-Tsoory and Mendelsohn, 2019; Osborne-Crowley, 2020).

It is important to note, however, Neisser’s call for more ecologically valid studies of cognition are not without detractors. Banaji and Crowder (1989) offered a rejoinder to Neisser’s arguments and argued that the ecological approach to cognition research is limited and scientific progress requires experimental control. Moreover, even the meaning of the term “ecological validity” has been criticized (Araujo et al., 2007; Dunlosky et al., 2009; Holleman et al., 2020; Schmuckler, 2001). Of note, a recent critique of the terminology of “ecological validity” was leveled by Holleman and colleagues (2020) who contend that prevalent conceptions of ecological validity are ill-formed, lack specificity, and fall short of addressing generalizability. They emphasize the need for more specificity in describing a given context in which cognition is being studied. Given this perspective, Parsons and colleagues (Parsons, Gaggioli, & Riva, 2020; Parsons & Duffield, 2020; see also Jolly and Chang, 2019) have reframed the discussion into considerations of low- and high-dimensional considerations of cognition. There are times when the parsimony offered by low-dimensional stimulus presentations may fall short of conveying the much higher-dimensional phenomena found in social, affective, and cognitive constructs. In fact, low dimensional stimulus presentations may at times offer diminished interpretations of complex phenomena.

### 1.2 Reframing the Ecological Validity Discussion into High-Dimensionality

**Figure 1:**
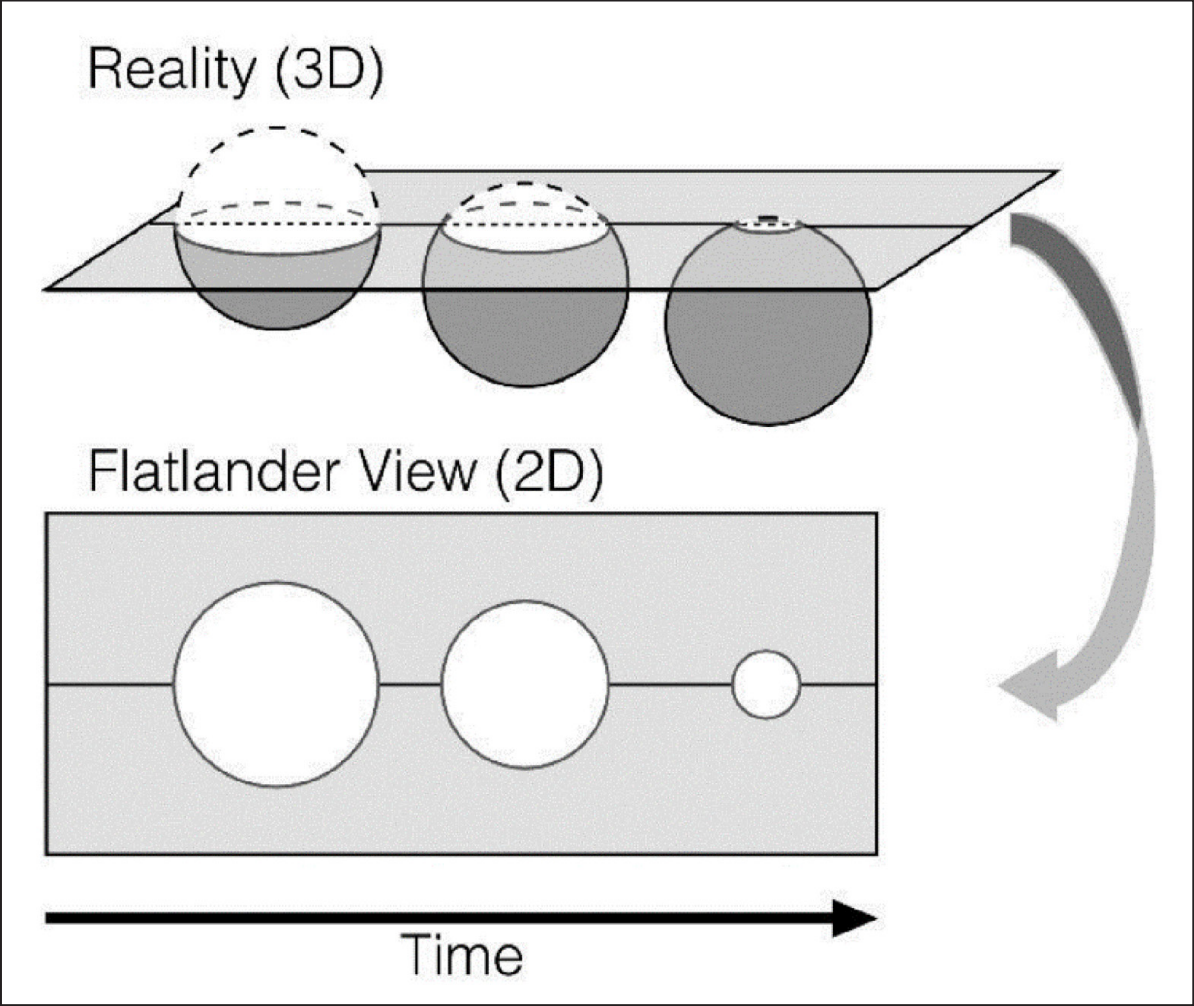
A. Square perceives his world as two-dimensional. (Reprinted by permission of the publisher).

The reframing of the ecological validity discussion into low-dimensional (static stimuli with limited to no interactivity using a mouse or keyboard; often 2D) and high-dimensional (dynamic, adaptive, and interactive 3D stimuli that require the naturalistic use of the upper limbs, head, and at times the full body) stimulus presentations can be likened to the Flatland perspective. In Edwin Abbott’s (1952) Flatland text on perception and dimensionality, the Flatlander A. Square (Abbot’s narrator) is only capable of perceiving two dimensions. A. Square encounters a “Stranger” (a sphere) who guides Square’s perspective into an understanding of the actual complexity (higher dimensionality) of the world. Likewise, in neuropsychology, low dimensional stimulus presentations may result in simplified explanations of complex phenomena, which may in turn limit the usefulness of models of human cognition, affect, and social interactions. Jolly and Chang (2019) call for psychologists to move beyond this “Flatland fallacy” via formalizations of psychological theories as computational models that can produce detailed neurocognitive predictions (see Figure 1).

The Flatlander’s constrained and low dimensional perspective (bottom of the figure) leads Square to perceive the three-dimensional sphere as a circle of varying sizes (increasing and decreasing radii). From the top of the figure, we can see that the object is a Sphere that is progressing across a lower-dimensional plane. The Flatland (low-dimensional) perspective limits the Flatlander’s perception and understanding of reality. Comparably, psychologists may at times fuzzily conclude that perceptions from a low level of dimensionality comprehensively explain cognitive, affective, and social phenomena.

The Flatland narrative calls attention to the need for developing models from high-dimensional stimulus presentations that better reflect the reciprocal relations among persons interacting with others in various environments (Beauchamp, 2017). This high-dimensional emphasis finds its roots in Ulric Neisser’s (1978) seminal work on ecological memory in psychological research. Neisser emphasized the need to move beyond narrow (low dimensional) laboratory investigations with limited generalizability to investigations involving activities of daily living. Likewise, researchers interested in cognition are arguing that an enhanced understanding of complex and dynamic interactions involved in the brain’s processes requires more complex stimulus presentations (Kennedy & Adolphs, 2012; Parsons, 2015; Wilms et al., 2010). Furthermore, there is an increasing realization of the need for developing high-dimensional tools that can be used for assessing and modeling brain functions (Parsons & Duffield, 2019; Zaki & Ochsner, 2009).

### 1.3 Extended Reality to Balance Ecological Validity with Experimental Control

What about the argument from Banaji and Crowder (1989) that Neisser’s arguments and the ecological approach to cognition research is limited and scientific progress requires experimental control? They argue that “…the multiplicity of uncontrolled factors in naturalistic contexts actually prohibits generalizability to other situations with different parameters” (p. 1189). A difficult issue for Banaji and Crowder’s argument is that while the controlled presentation of everyday objects as low-dimensional pictorial stimuli does allow for rigorous control, the meaningfulness of findings is obscured as cortical object processing does not involve processes entirely invariant to an object’s physical properties (Holler et al., 2020). Cognitions (e.g., memories) found in conventional laboratory events inadequately reflect engrams formed under realistic (complex) conditions (Cabeza et al., 2004; Cabeza and St Jacques, 2007).

It appears that a tertium quid is needed that balances the needs for ecological validity and experimental control. Here is where the high-dimensional Metaverse comes into play. The Metaverse is an extended reality of the Internet that provides cognition researchers with a variety of virtual, augmented, and mixed-reality experiences (Mystakidis, 2022). Technological enhancements found in high-dimensional Metaverse experiences hold promise for enhancing studies of cognition. While neurocognitive researchers are increasingly moving away from low-dimensional approaches (Hasson and Honey, 2012; Maguire, 2012; Hamilton and Huth, 2018; Matusz et al., 2019; Sonkusare et al., 2019), high-dimensional stimulus presentations like the ones found in extended reality (e.g., virtual/augmented/mixed-reality) are needed because they offer enhanced stimulus presentations and interactivity. To clarify, extended reality (XR) is a collective term for immersive technologies that include: (1) virtual reality (VR) which fully immerses the participant into a computer-generated virtual world; (2) augmented reality (AR) which overlays digitized content onto the real world; and (3) mixed reality (MR) which includes virtual objects that are not just overlaid on the real world but can interact with it. It is important to note that just like explanations of technological innovations are constantly evolving, what defines XR is consistently developing (Palmas & Klinker, 2020). Observations of persons interacting in extended reality simulations that reflect activities of daily living may refine previous understandings of cognition.

The metaverse offers enhanced control of the various extraneous variables that can be problematic (even impossible) for experimental control in naturalistic observation studies (Sauzéon et al., 2015; Dombeck and Reiser, 2012; Parsons, 2015; Parsons, Gaggioli, & Riva, 2020). Virtual reality studies reveal successful facilitation of the formation of profound memory traces (Kisker et al., 2021a, 2021b; Schöne et al., 2019). This suggests that the metaverse technologies like virtual reality weave experiences into a participant’s narrative or autobiographical memory in a manner similar to everyday activities. As a result, metaverse platforms may offer enhanced research into the neural correlates of encoding for manipulations not appropriate for execution in a real-life context (e.g., Bréchet et al., 2019; Vass et al., 2016).

## 2 Algorithmic Devices and Digital Extension of Cognition

In addition to the enhanced stimulus presentation found in these high-dimensional XR platforms, adaptive algorithms and wearable sensors may extend users’ cognitive, affective, and social processes beyond the wetware of their brains. This is apparent in Metaverse technologies that enable us to navigate, translate, recall, analyze, and compute information via adaptive extended reality environments that use machine learning to personalize the user’s experience.

### 2.1 Cognitive Offloading and Extended Cognition

According to Daniel Dennett (1996), the human brain regularly offloads cognitive tasks into the environment by

off-loading as much as possible of our cognitive tasks into the environment itself – extruding our minds (that is, our mental projects and activities) into the surrounding world, where a host of peripheral devices we construct can store, process and re-represent our meanings, streamlining, enhancing, and protecting the processes of transformation that are our thinking. This widespread practice of off-loading releases us from the limitations of our animal brains. (pp. 134–135)

This suggests an active externalism in which cognitive processes are interactively being performed by our brains and the technologies we use (Clark, 2008; Clark & Chalmers, 1998). According to Andy Clark (2008), human brains may initiate extra-organismic resources into problem-solving, thereby “creating hybrid cognitive circuits that are themselves the physical mechanisms underlying specific problem-solving performances” (Clark, 2008, p. 68). A “parity principle” can be used for analyzing the extension of extended cognitive systems from brain-based cognitive processes to external objects (e.g., metaverse technologies):

If, as we confront some task, a part of the world functions as a process which, were it to go on in the head, we would have no hesitation in recognizing as part of the cognitive process, then that part of the world is (so we claim) part of the cognitive process. (Clark & Chalmers, 1998, p. 8)

The parity principle is optimally considered a heuristic device for evaluating the applicability of presumed cases of cognitive extension. Of note, Clark (2011) suggests that the parity principle is intended:

to invite the reader to judge various potential cognitive extensions behind a kind of ‘veil of metabolic ignorance’. A good way to do this is ask yourself, concerning some candidate cognitive process P, whether if you were to find P (or better, its functional equivalent) occurring inside the head of some alien organism, you would tend to class P as a cognitive process? (Clark, 2011, p. 449)

Clark and Chalmers employ fictional characters, Inga and Otto, who must navigate to the Museum of Modern Art (MoMA) on Fifty-Third Street in New York City. Inga can readily recall the directions to the MoMA from her internal brain-based cognitive processes. For Otto, things are different. He is in the early stages of Alzheimer’s disease and this limits his cognitive capacities for recalling the directions from sole use of his internal brain-based cognitive processes. As a substitute, Otto supplements his brain-based cognitive capacities with an external aide found in a notebook. Here, the brain and notebook are coupled in an information-processing loop that extends beyond the neural realm to include elements of Otto’s environment.

The cases of Otto and Inga illustrate that mental processes cannot be fully reduced to brain processes. Take, for example, the potential of smartphones connected to the Internet to extend our brain-based memory. Mobile technologies connected to the Internet allow for novel investigations into the interactions of people as they engage with a global workspace and connected knowledge bases. Moreover, mobile access to the Internet may allow for interactive possibilities: a shift in how we see ourselves and the ways in which we understand the nature of our cognitive and epistemic capabilities (Parsons, 2017).

### 2.2 Metaverse as Candidate Cognitive Process for Extension

If we take the Metaverse as our case, the “candidate cognitive process” might be extended reality-based cognitive assessment processes. Consequently, the performance request is that one imagines a state of affairs in which some everyday cognitive task is being performed inside the brain of a given user (i.e., without the use of a head-mounted display). If one is inclined to accept the cognitive status of the everyday cognitive task when it is performed inside the brain of an individual, then the claim is that one should accept the cognitive status of the everyday cognitive task when it is subject to an alternative form of mechanistic realization. Take, for example, the potential of virtual reality-based cognitive assessments connected to the Internet to extend our brain-based cognition. Metaverse virtual reality-based cognitive assessment technologies connected to the Internet allow for novel investigations into the interactions of a person’s cognitions as they engage with a global workspace and connected knowledgebases. Moreover, metaverse access of virtual reality-based cognitive assessments to the Internet may allow for interactive possibilities: a shift in how we see cognitions and the ways in which we understand the nature of our cognitive and epistemic capabilities (Parsons, 2017).

Cognition is further extended in the metaverse with the addition of algorithmic rules, strategies, and procedures that a person can use to aid cognition and problem-solving. Reiner and colleagues have termed these algorithmic devices as technologies of the extended mind (Fitz & Reiner 2016; Nagel & Reiner, 2018; Reiner & Nagel, 2017). A technology of the extended mind (TEM) acts as a relatively continuous interface between brain and algorithm such that the person experiences the algorithmic device as an extension of the person’s mind:

It is not the case that every algorithmic function carried out by devices external to the brain qualifies them as a TEM, but rather that there is a relatively seamless interaction between brain and algorithm such that a person perceives of the algorithm as being a bona fide extension of a person’s mind. This raises the bar for inclusion into the category of algorithms that might be considered TEMs. It is also the case that algorithmic functions that do not qualify as TEMs today may do so at some future point in time and vice versa. (Reiner & Nagel, 2017, p. 110)

This addition to Clark and Chalmers’s parity principle specifies the features needed for a technology to be an extension of a person’s mind. They emphasize that the concept of an extended mind requires the presence of a relatively seamless interaction between the person’s brain and the algorithm such that the person perceives the algorithm as a bona fide extension of their mind. The advent of the metaverse, social virtual reality, and the Internet of Things (IoT) connects everyday objects (including virtual simulations) via algorithms to the Internet and enables data transfer from network-connected devices to remote locations. The rise of telepsychology and the Virtual Environment of Things (VEoT) extends the user’s experience of real-world smart technologies with virtual objects and avatars in interactive and adaptive virtual environments (Lv, 2020; Wu, Chou, & Jiang, 2014).

## 3. A Neurocognitive Framework for Understanding Technologies of the Extended Mind

A neurocognitive framework for understanding technologies of the extended mind has been proposed by Parsons (2017, 2019). This approach builds on Stanovich’s (2009a) tripartite model of cognitive processing which includes an autonomous (automatic; rapid; nonconscious use of heuristics) processor and a controlled processor (slow; effortful, largely conscious) with two subdivisions: (1) reflective processing characterizes the goals of cognitive processing, goal-relevant beliefs, and optimizing choices of action; and (2) algorithmic processing that includes “mindware” that consists of the rules, strategies, and procedures that a person can retrieve from memory to aid problem-solving. Neurocognitive support for this tripartite model comes from three neural systems (see Wood and Bechara, 2014): (1) automatic processing (fast, automatic, nonconscious, and habitual behaviors) via amygdalastriatal (limbic-ventral striatal loop structures such as ventral striatum and amygdala) system; (2) reflective processing (planning, prediction, and inhibitory control) via prefrontal-dorsal striatal loop (prefrontal cortex mediation of decision making and inhibitory control); and (3) algorithmic interoception via the insular cortex. The limbic-ventral striatal loop (automatic) and prefrontal-dorsal striatal loop (reflective) processing can be assumed to act in parallel and can interact with each other during decision-making, whereby one system acts in a predominant role. Situational and environmental features influence the processes that activate a predominant system (Schiebener and Brand, 2015). The insular cortex offers a third (interoceptive awareness) system that activates representations of homeostatic states to translate somatic states into more conscious states (Noël et al., 2013) and modifies the equilibrium between the automatic and reflective system (Wood and Bechara, 2014).

### 3.1 Neuropsychologically-Based Tripartite Processing Model of Technologies Extending Cognition

**Figure 2:**
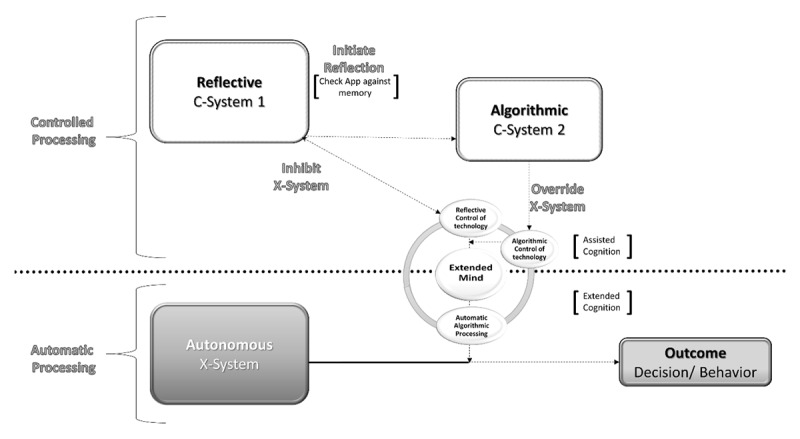
Framework for understanding technologies of the extended mind. Note that the dividing line between Controlled and Automatic Processes reflects the level of conscious awareness of the user. The X-System is nonconscious and autonomous. Whereas C-System 1 (and to some extent C-System 2) are largely conscious processes that require greater effort and attention.

Parsons (2019) has combined this work and developed a neuropsychologically-based tripartite processing model of technologies that extend cognition (see Figure 2). According to Parsons’s framework and model 1) automatic algorithmic processes originating with an algorithmic device are coupled with the automatic (X-System) processing of the limbic-ventral striatal loop, 2) reflective (C-System 1) of the prefrontal-dorsal striatal loop, and 3) algorithmic (C-System 2) of the insular cortex. The algorithmic processes of human-technology interactions can, over time, become an automated and algorithmic coupling of brain and technology. When the user first starts operating a new device, there is a period in which the user relies on controlled (C-System 1: reflective) cognitive processes found in the prefrontal-dorsal striatal loop to inhibit and override prompts initiated by the device (see reflective and algorithmic control of technology in Figure 2). After using the technology for a period of time, the algorithmic operations become overlearned and more or less rely automatically upon the limbic-ventral striatal loop (X-System: automatic processing). The extension of these brain processes to algorithmic technologies is balanced (C-System 2) by insula cortex processing of salient environmental factors that bias a technology user’s deployment of automatic (X-System: limbic-ventral striatal loop) and reflective (C-System 1: prefrontal-dorsal striatal loop) information processing.

### 3.2 Neurocognitive Approaches to Presence and Embodiment

The coupling of human brains to algorithmic technologies found in the Metaverse involves embodiment, which includes a sense of self-location and agency “as if” the user were in the real world instead of the metaverse. Through a combination of embodiment, immersion, and interactivity metaverse platforms create an “as if” sense of presence that allows the user to feel and behave as if they were in the real world (Riva, 2022). Feeling a sense of presence, the user acts “as if” virtual objects and avatars are real objects and agents. This embodiment and “as if” sense of presence result from a coupling of algorithmic metaverse simulations and the predictive coding of the user’s brain. Predictive coding in neuroscience refers to how one’s brain actively creates an internal model (embodied simulation) of the user’s body and surrounding environment (Friston, 2010, 2012, 2018; Clark, 2013) that is used for both predictions about anticipated sensory inputs and prediction error minimization (Talsma, 2015). According to Riva and colleagues (2019), embodiment in metaverse technologies involves sensory-motor experiences that use visceral (i.e., interoceptive), motor (i.e., proprioceptive), and sensory (e.g., visual, aural, kinesthetic, olfactory, gustatory) information as sources. Moreover, embodiment balances these information sources with the user’s multimodal neural networks. Metaverse technologies (e.g., virtual reality) use artificial neural networks, as well as machine learning-based classifiers and predictors to anticipate the sensory outcomes of a user’s actions by presenting the user immersed in a virtual world with the outcomes anticipated by the user’s brain in the real world.

Situating presence within Parsons’s model involves looking to Hartmann’s (2015) postulation that the experience of presence is primarily established in X-System’s automatic and heuristic response style and rapid subconscious processing. Moreover, Hartmann assumes that there is less involvement from the controlled processor (C-System 1; C-System 2) as it handles more focused cognitive processes. Fitting this into Parsons’s theoretical framework, the evolutionary older X-System processes the virtual environment’s information rapidly, easily, and unconsciously. This gives rise to the feeling of “being there” (i.e., sense of presence). Moreover, the evolutionarily younger controlled processing C-System 1 gives rise to beliefs about the user’s experience that are given weights (somatic markers biasing future decisions) by C-System 2 about the virtual reality experience. Given that these systems operate in parallel, users immersed in a virtual environment may simultaneously feel spatially present while recognizing they are not. This leads to Metaverse platforms that can offer impactful experiences for users because the user experiences virtual rewards and penalties as if they were real. Despite being virtual, these reinforcement schemas are difficult to resist. Virtual environment and videogame developers use these inducements to ensure that users (e.g., videogamers) keep using the algorithmic devices (e.g., virtual environments; video games).

### 3.3 The Automatic X-System: Dopaminergic Reward Systems and the Amygdala

The automatic (X-System) system includes the striatum (dopaminergic reward systems) and the amygdala, which mediate reward-seeking and compulsion, through sensitization (Noël, Brevers, & Bechara, 2013). This limbic-ventral striatal loop (X-System: automatic processing) system has been found to be sensitive to coupling with algorithmic devices (e.g., playing videogames in the metaverse). For example, positron emission tomography has revealed that substantial use of the metaverse (e.g., playing online games) is associated with synaptic structure plasticity and dopamine availability in striatal regions (Hou et al., 2012; Kim et al., 2011). Likewise, Internet gaming research using voxel-based morphometry has found that greater metaverse gameplay is associated with increased left striatal and right caudate volume (Kühn et al., 2011; Cai et al., 2016), as well as lower bilateral amygdala gray matter density (Ko et al., 2015). Additionally, the repeated coupling of brains and technologies (e.g., playing videogames) strengthens the association between technology use and reward (Turel, Serenko, & Giles, 2011). In terms of presence, findings of Jäncke and colleagues (2009) suggested a distributed presence network that includes the visual stream (dorsal and ventral), the parietal cortex, the premotor cortex, the mesial temporal areas (hippocampus, amygdala and insula), the brainstem, and the thalamus. It is important to note that while X-System does not include the insula (C-System 2), there are clear projects from X-System to C-System 2.

### 3.4 The Reflective C-System 1: Prefrontal-Dorsal Striatal Loop

The reflective (C-System 1: prefrontal-dorsal striatal loop) system controls working memory and executive functions (e.g., inhibition of prepotent responses, mental set shifting). These controlled cognitive processes are primarily dependent on the prefrontal cortices and the anterior cingulate cortex. The algorithmic dependence that occurs in excessive metaverse videogame play is associated with decreased functional connectivity in the prefrontal cortex (Jeong et al., 2016; Jin et al., 2016) and significant hyperactivity in the anterior cingulate cortex (Ding et al., 2014). Moreover, reduced fractional anisotropy in the dorsolateral prefrontal cortex and anterior cingulate cortex has been found in excessive metaverse gaming (Yuan et al., 2016). As a result, excessive use of technologies impacts brain areas responsible for the critical abilities of the reflective system to suppress cognitive (Cai et al., 2016; Ko et al., 2014) and motor response inhibition (Ding et al., 2014). In addition to these neuroimaging studies that consistently report abnormalities in brain structure and function in Internet gaming disorder, a number of quantitative meta-analyses have synthesized the literature (Niu et al., 2022; Solly et al., 2022; Sun et al., 2023) and revealed structural and functional impairments in brain regions related to executive cognitive control. In terms of presence, greater levels of presence were associated with smaller activation in the dorsolateral prefrontal cortex (Jäncke et al., 2009).

### 3.5 The Algorithmic C-System 2: Insular Cortex

The algorithmic (C-System 2: interoceptive awareness) system relies on the insular cortex (insula). The insula is understood to be a gateway to visceral needs and mediates the generation of homeostatic perturbations (Craig, 2009; Zhao et al., 2023). The insular activity found in C-System 2 can stimulate motivation by biasing effective incentive inputs to feedback loops (Noël et al., 2013). The insula plays a role in excessive metaverse technology use (e.g., Internet gaming disorder) and neuroimaging studies have found decreased functional connectivity between the insula and the motor/executive cortices (prefrontal cortex; cingulated cortex) found in the reflective (C-System 1) system (Zhang et al., 2016). Furthermore, neuroimaging during metaverse-based videogame-related cues reveal evidence suggesting robust associations between the insula and the automatic (X-System) and reflective (C-System 1) systems (Ko et al., 2009). Activation of the insula has been associated with the generation of presence as it plays a role in the user’s sense of self-awareness and body-ownership. This leads to the formation of the user’s “body schema” and increases their feeling of embodiment while immersed in a virtual environment (Baumgartner et al., 2006; Clemente et al., 2014; Jäncke et al., 2009).

### 3.6 An Update to the Parity Principle

To elucidate Parsons’s tripartite neuropsychological conceptualization, it is helpful to consider Reiner’s update to the parity principle that emphasizes the need for a reflective (C-System 1) perception of the coupling with an algorithmic device. This can be illustrated via a participant named Inga who is taking part in a metaverse experiment in Tempe Arizona. Inga’s use of a Metaverse avatar allows her to virtually experience a high-dimensional 3D rendering of the Museum of Modern Art (MoMA) “as if” she were actually in Midtown Manhattan, New York City (NYC). The metaverse avatar offers Inga an embodied and multisensory virtual presence experience of a metaverse-based museum. Inga puts on and wears a head-mounted video display that allows her to see, hear, and smell (via a smell machine). Inga is also wearing haptic gloves and a body suit that delivers haptic feedback while capturing motion and biometrics. While immersed and embodied in the metaverse, Inga is able to navigate, interact, and move anywhere in the Museum utilizing a completely remote-controlled avatar. The metaverse platform includes an omnidirectional treadmill, as well as integrated visual, audio, and haptic presentation of stimuli in an interactive environment. This allows Inga to freely move between exhibit locations, at Inga’s own pace.

After being immersed in a metaverse simulation of the MoMA, Inga can navigate using an application that offers coordinates similar to those found via GPS coordinates at the MoCA in NYC. The application also includes a set of machine learning algorithms that allow it to take logged behaviors and biometrics to learn her preferences. Inga has read the manual that came with the Metaverse platform and understands that she can search for exhibits by entering them into the Metaverse avatar app which will show her the best route to displays. Once Inga arrives at her target location, Inga can interact with the Metaverse avatar app to learn about the exhibit. This is seen as a benefit by Inga because allows her to keep losing her way when exhibits are in unfamiliar parts of the museum. At the start, the machine learning algorithms knew little about Inga. This lack of adaptivity from the algorithmic processing of Inga’s behavioral and biometric data, plus Inga’s limited familiarity with this application, results in some initial skepticism about the technology. As a consequence, Inga continues to be on the alert (see controlled/reflective processing of C-System 1(prefrontal-dorsal striatal loop) in Figure 2) to her environment so that she can be sure that she makes it to the museum exhibits without any problems.

Putting this back into a neurocognitive framework, Inga’s initial reliance is on C-System 1 (prefrontal-dorsal striatal loop) and she remains alert to potential limitations and double-checks the veracity of feedback (somatic markers) from C-System 2 from the metaverse avatar Museum application. After sustained effective use Inga basically accepts the Metaverse avatar app and only occasionally stops herself from automatically (X-System: limbic-ventral striatal loop) following the application’s guidance (see inhibition and override of technology using reflective and algorithmic control of technology in Figure 2). Is Inga’s metaverse avatar app functioning as a technology of the extended mind? While it is indeed performing computations that are external to Inga’s brain, the functions of the Metaverse avatar app are perhaps more optimally conceptualized as cognitive assistance. The reason is that neither the algorithmic calculations from the Metaverse avatar app nor Inga’s use of them are automated with Inga’s cognitive processes (see algorithmic control of technology in Figure 2).

Changing the scenario somewhat, what if Inga has experienced the Metaverse exhibits several times over the course of a month? Despite now having slightly more knowledge of the Metaverse museum, she relies on her Metaverse avatar app to navigate through the museum. The Metaverse avatar app has not failed her in its directions to exhibits or its information (e.g., artist, history, subtleties of the work) about the art at each exhibit. Each time Inga enters an exhibit into the Metaverse avatar app’s search interface and the route is presented on the screen of the head-mounted display, she automatically (X-System: limbic-ventral striatal loop) follows it to the destination suggested and readily receives information about the art. The metaverse avatar app is beginning to function as a technology of the extended mind because Inga has integrated its algorithmic processes into the workings of her mind.

## 4. Conclusions: High-Dimensional Metaverse as Rapprochement

In conclusion, there is a growing desire for a balance of experimental control and ecological validity. Experimental control has largely resulted in low-dimensional stimulus presentations that may at times offer diminished interpretations of complex phenomena. Moreover, the limitation of using low-dimensional pictorial stimulus presentations of everyday objects to optimize control is the meaningfulness of findings—as cortical object processing does not involve processes entirely invariant to an object’s physical properties (Holler et al., 2020). Cognitions (e.g., memories) found in conventional laboratory events may inadequately reflect engrams formed under realistic (complex) conditions (Cabeza et al., 2004; Cabeza and St Jacques, 2007). That said, the term “ecological validity” has been criticized (Araujo et al., 2007; Dunlosky et al., 2009; Holleman et al., 2020; Schmuckler, 2001).

There is a need for more specificity in describing contexts in which cognitions are being studied. Given this perspective, some (Jolly and Chang, 2019; Parsons, Gaggioli, & Riva, 2020; Parsons & Duffield, 2020) have reframed the discussion of ecological validity into considerations of low- and high-dimensional stimulus presentations and the extent to which embodied coupling of humans and technologies can extend cognition. There are times when the parsimony offered by low-dimensional stimulus presentations may fall short of conveying the much higher-dimensional phenomena found in social, affective, and cognitive constructs. In fact, low dimensional stimulus presentations may at times offer diminished interpretations of complex phenomena.

The Metaverse has the potential to extend our cognitive processes beyond the wetware of our brains. A way of considering this issue is to consider the mind as representing the full set of cognitive resources that the person deploys in the service of thinking. Thinking can be understood as automatic, reflective, and algorithmic (Stanovich, 2009a, 2009b; Parsons, 2019). This approach comports well with the extended mind hypothesis because the idea of a “full set of cognitive resources” allows for additional contributions (in addition to the brain) to conceptions of mental processing.

The extension of mental processes outside of the brain (e.g., technologies of the extended mind) means that mental processes cannot be fully reduced to brain processes. Take, for example, the potential of Metaverse avatar app connected to the Internet to extend our brain-based cognitions (e.g., navigation, memory). The coupling of the brain and the Metaverse avatar app not only enhances the user’s cognitive capacities but also moves the technologies beyond memory assistants to powerful Metaverse simulations. In fact, Metaverse avatar app technologies connected to the Internet allow for novel investigations into the interactions of persons as they engage with a global workspace and connected knowledgebases. Moreover, the Metaverse may allow for interactive possibilities: a paradigm shift in how we see ourselves and the ways in which we understand the nature of our cognitive and epistemic capabilities (Parsons, 2017).

Using high-dimensional metaverse technologies of the extended mind, ecological validity could be operationalized as the extent to which the results of a Metaverse study can be generalized to real-life settings. Hence, ecological validity would be focused on whether the findings of a Metaverse MoMA study can be generalized to naturalistic situations, such as visiting the MoMA on 53^rd^ Street in Midtown Manhattan. The ecological validity of the Metaverse MoMA experience could be calculated as a correlation between ratings obtained with the Metaverse MoMA experience and an appropriate measure in naturalistic visits of the MoMA in New York.

To illustrate this idea, we could take the case of Inga immersed in the Metaverse MoMA (while online in Arizona), and Junior (Otto’s son) at the New York MoMA. We could record the behavioral and biometric data from both Inga’s (Metaverse MoMA while in Arizona) and Junior’s (New York MoMA) experience and compare them. In fact, we could do a randomized controlled trial with enough participants to make sure that it has adequate statistical power. A large-scale trial like this would give us a good idea of the Metaverse MoMA’s ecological validity. Participants would be randomized to one of two counter-balanced MoMA order conditions: 1) a traditional visit to MoMA in New York, or 2) a Metaverse MoMA experience followed by a traditional in-person visit. Permuted block randomization could be used with randomly varying block order and length. After randomization to modality (NYC MoMA or Metaverse MoMA) order, participants would be contacted by a research coordinator to schedule the two experiences (perhaps 30 days apart). Pearson correlations could be used to determine the relations between the behavioral and biometric data from Metaverse MoMA and the corresponding in-person New York MoMA experience (convergent and divergent validity).
